# The 100 most cited articles in peri-implant diseases. A bibliometric study of the web of science

**DOI:** 10.4317/jced.59786

**Published:** 2022-11-01

**Authors:** Andrés López-Roldán, Beatriz Tarazona-Álvarez, Antonio Vidal-Infer, Adolfo Alonso-Arroyo

**Affiliations:** 1 Department of Stomatology. School of Medicine and Dentristy. University of Valencia, Spain; 2 Department of History of Science and Information Science. School of Medicine and Dentristy. University of Valencia, Spain; 3 UISYS, Joint Research Unit (CSIC – University of Valencia)

## Abstract

**Background:**

The purpose of the study was to analyze the 100 most-cited articles on peri-implantitis pathology in the Web of Science database.

**Material and Methods:**

The articles were selected from all categories of the Web of Science, to consider all variations and synonyms of peri-implant disease. Articles were reviewed for typographical, transcription, and indexing errors.

**Results:**

The top 100 most-cited articles were published from 1994 to 2018, and had a total of 24,103 citations; 53 of the studies were funded. In total, 274 authors contributed to the papers, 5 of whom contributed to 10 or more articles. Review (n = 47) and clinical (n = 45) articles were the most prevalent types. European public universities made the largest contribution to the literature, and Sweden and Switzerland were the most active countries. All of the articles were published in 12 high-impact-factor journals.

**Conclusions:**

This is the first analysis of the most-cited articles on periimplantitis published in the Web of Science. In this bibliometric analysis, the most cited articles were published in high-impact-factor journals and were predominantly review articles. The most-cited authors are also active in other scientific disciplines such as periodontics.

** Key words:**Dental implantation, Dental implants, Peri-implantitis, Mucositis.

## Introduction

Bibliometric analysis provides information on the most productive authors, institutions, and countries for a given field of research, based on the number of scientific publications indexed in a database and, where possible, on the number of citations (1). Although not necessarily indicative of the quality of an article (2), the number of citations, as well as the impact factor of the journal in which it is published, are used to assess the academic status of researchers (3). Widely cited articles are referred to as top cited or hot papers.

Peri-implant diseases are among the most frequent causes of dental implant problems and decrease the likelihood of success of implant treatment. These diseases have emerged relatively recently, because dental implant therapy started only four decades ago (4,5). Research is required to improve the clinical outcomes of dental implants. Therefore, peri-implant disease is a frequent topic among the most-cited articles on dental implantology (6).

Bibliometric studies have analysed the most cited articles in dentistry (6-8) and in subspecialties such as endodontics (9-11) orthodontics (12) and periodontics (13). There are also bibliometric studies on implantology (6,14). Nevertheless, there are no bibliometric studies on peri-implant pathologies has been performed. These investigations have progressively increased from 5.5% to 13.6% in the last 20 years (15).

To address the lack of bibliometric studies in the Peri-Implant diseases field, we performed a bibliometric study of the 100 top-cited articles on peri-implant diseases.

## Material and Methods

The most cited articles on peri-implant diseases were identified by searching the Science Citation Index Expanded database of the Web of Science (Clarivate Analytics). The search terms were as follows: (peri-implantitis OR periimplantitis OR periimplantitis OR peri-mucositis OR “Implant* mucositis” OR “peri-implant* disease*” OR “periimplant* disease*” OR “peri-implant* mucositis” OR “periimplant* mucositis”); these terms were entered in the ‘topic’ field, with no restrictions regarding the year of publication. Limitations were imposed on the type of document (article OR review). The search was performed on 21 February 2022. The results were sorted according to the number of citations per article, and the first 200 records registered were selected and exported to a text file. This text file was imported from Microsoft Access (Microsoft Corp., Redmond, WA, USA) to create a database. A manual review was performed, and articles found not to be relevant were discarded. Only the top 100 most-cited articles were included in the analysis.

Subsequently, terms were unified, and t typographic and indexing errors removed. Firstly, The records were manually normalized; whenever it was not clear whether different authors shared the same name, their institutional affiliation was investigated. Similar criteria were applied to normalise institutional data; macro-institutions (universities, hospitals, etc.) were retained, while departments, research units, etc. were removed.

The variables analysed included the journal in which the article was published, year of publication, authors, institutions, country of origin, and topic. Ethics approval was not required for this bibliometric study.

## Results

Table 1 lists the study designs. Of the 100 articles, 28 were reviews, of which 5 were consensus documents; there were 19 systematic reviews, including 3 meta-analyses (1 consensus document). The reviews had the most citations , but systematic reviews had the highest average number of citations per article. Notably, *in vitro* studies had a high number of citations per article.

**Table 1:**
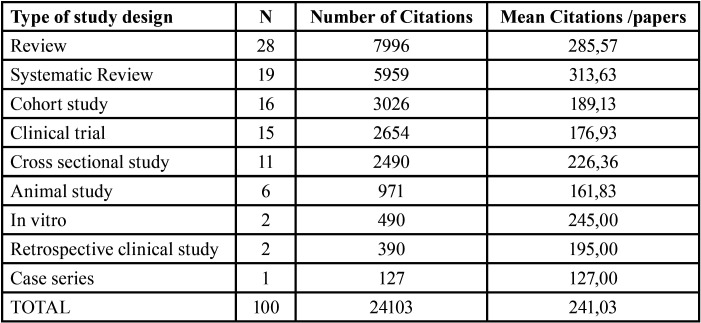
Total citations and mean citations/paper by type of study design.

Data recording began in 1994; 2012 was the most productive year (12 articles published), but 1998 and 2008 were also productive. The number of citations was highest during the 3-4-year period after publication.

A total of 274 authors contributed to the studies, of whom 94 have only published 1 paper and 5 have published 10 or more papers. Niklaus Lang was the most productive author (19 articles among the 100 most cited) and has the highest total number of citations (Table 2). However, the author with the highest average number of citations per article is Marco Esposito. There was a relationship between the number of publications and number of citations in some, but not all, cases. The average number of citations per article was correlated with productiveness, i.e. the total number of citations.

**Table 2:**
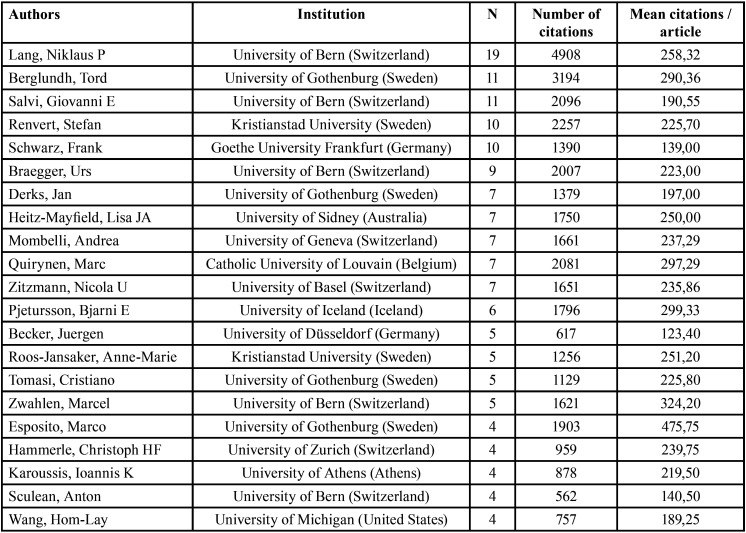
Ranking of authors and last affiliation. Sorted by production, together with the values of the number of total citations and average citations per article.

The authors were mainly European, with the most frequent affiliation being to a Swiss university. However, the data only included the last affiliation, and some authors have worked at various institutions during their professional career .

A total of 98 institutions participated in the studies, of which the majority were universities (n = 86), followed by hospitals/clinics (n = 9), research groups (n = 2), and government institutions (n = 1).

All 25 institutions with the highest total number of citations were universities (Table 3). European universities topped the list, with Sweden and Switzerland being the best-represented countries. The institution with the most citations was the University of Gothenburg.

**Table 3:**
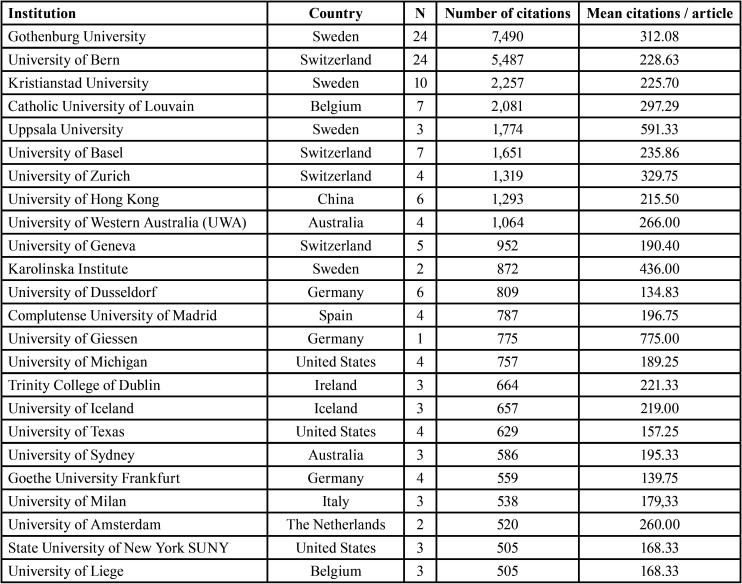
Ranking of the 25 institutions with the highest number of total citations.

Countries with more than 5 articles among the top 100 were European countries, although the United States was in third position, while Australia was in fifth and China in ninth. Thirty countries participated in the publications, led by Sweden (21.19% of all publications). The top three countries contributed 53.82% of the publications, and the remaining 27 countries contributed 46.18%.

There was no direct correlation between the number of authors and total number of citations or citations per article. The citation rate was higher for national collaborations, at 289.81 citations per article.

Of the 100 articles, 53 were funded by 67 funding institutions in 17 countries; these institutions were companies, governments, academic institutions (public universities, private universities, and research institutes), and non-profit entities (foundations and scientific societies). The best-funded countries were Sweden (governmental and non-profit institutions) and the United States (private companies) (15 articles each). Regarding the types of funding institutions, 23 were companies, 18 were non-profit entities, 15 were academic entities and 11 were government institutions. However, the order changes if the number of funded publications is considered: non-profit entities funded 25 articles, academic entities 20 articles, companies 17 articles, and government entities 16 articles. Table 4 shows the best-funded institutions among those with more than two funded articles; the Swiss International Team for Implantology is top of this list.

**Table 4:**
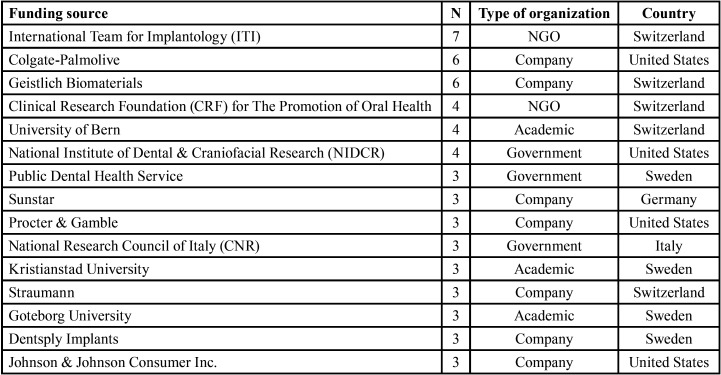
Financing sources of more than 2 publications.

The 100 most-cited articles on peri-implant pathologies were published in 12 journals. All of those journals have a high impact factor, i.e., are in the top two quartiles, and the vast majority are in the Dentistry, Oral Surgery and Medicine category. Of the 100 articles, 44 were published in Clinical Oral Implants Research. The two works published in the European Journal of Oral Sciences had an average of 790 citations.

The most frequently used were peri-implantitis and dental implant. Keywords directly related to peri-implant diseases included peri-implant disease (ranked third), peri-implantitis (ranked first), and peri-implant mucositis (ranked sixth). One group of keywords was directly related to implant success (failure, survival rate, and success rate), another to complications (biological complications and other complications), and a third to the aetiology of peri-implant pathologies (dental plaque and bacteria).

The articles discussed diverse aspects of peri-implant pathologies, but particularly treatments, followed by epidemiology, risk factors, and microbiology. Overall, 69 articles focused on peri-implant pathologies, 18 on peri-implantitis, and only 3 on peri-implant mucositis.

## Discussion

Peri-implant pathologies have aroused scientific interest because of their clinical implications and association with failure of a previous treatment, such as dental implantation. This interest is reflected in our analysis of the 100 most-cited articles in this area. Such articles first appeared in 1994, but were most prevalent in 2012. These years coincide with publications from a consensus meeting on dental implants. All of the articles were published in high-impact journals, indicating that editors are interested in this topic. The impact factor is linked to the number of citations and vice versa, so publishing in high-impact journals is desirable (3).

The most-cited articles were reviews and systematic reviews, as seen in other disciplines (16,17), and particularly in the dental literature (6,7,13) . This is attribuTable in part to the frequent citation of the latest revision of an article instead of the original (18), which is known as lazy author syndrome (19). Consensus documents derived from workshops are also important; these are documents based on reviews that include conclusions by experts. The high citation rate for *in vitro* studies that we observed is interesting, and may reflect the importance of material and mechanical factors in periimplant diseases.

Publishing more articles increases an author’s total number of citations, as in the case of Niklaus Lang. However, if the average number of citations per article is considered, the rank order changes. Many authors are cited in other areas of dentistry, such as implant therapy (14) and periodontics (20).

Niklaus Lang’s top ranking is attributable to his focus on research on peri-implant pathologies at some of the top-ranked institutions, such as the University of Bern and the University of Gothenburg. He also serves as an editor of several journals.

Reviews were the most frequently cited articles. Some reviews are commissioned, and authorship may be influenced by the so-called Saint Mathew’s effect (21) , where experts in the field are often the authors of the reviews.

The vast majority of institutions in this study were universities, similar to prior bibliometric studies related to implants (14) , but not to other medical disciplines in which hospitals are dominant (22).

The most prolific universities tended to be European, especially Swedish and Swiss ones, represented by the University of Gothenburg and University of Bern, respectively. Both of these institutions were ranked highly for total citations. This trend has also been observed in bibliometric studies of implantology (6), and may be attributable to modern implantology originating from this region (4,5). The level of funding is also high in these countries. In summary, European countries and the US were the most scientifically productive regions, as in other areas of dentistry (6,14).

More than half of the articles were funded, similar to dental implantology, although most of the financing entities were corporate rather than governmental (23). The entities funding implantology studies were similar to those funding peri-implant pathology studies. Most institutions were based in Switzerland, Sweden, or the US, i.e. in the countries with the highest number of total citations. There was a tendency for the funding for studies to emanate from the same country, and funding also seems to influence the number of citations.

Dentistry journals had the most cited articles, as in periodontics (24) and implantology (14). Clinical Oral Implant Research and the Journal Clinical of Periodontology accounted for 39.8% and 29.8% of all citations, respectively, i.e. 69.6% of the total citations. The European Journal of Oral Sciences had very high average numbers of citations per article, and the first and third most-cited articles. In fact, these articles are from the same review on biological factors contributing to implant failure, both authored by Marco Esposito.

The most used keyword was peri-implantitis. This biological complication was of interest in other bibliometric studies (6). The most-cited articles were on peri-implant pathology, epidemiology, risk factors, treatment, and microbiology, as in bibliographic studies on periodontal pathology (13).

This study had several limitations that should be discussed. Firstly, the truncated term “periimplant*”, and similar words, were used as search terms, but some articles may have been missed since the words had to be included in the “Subject” field (Title, Abstract and keywords). The search identified several articles that did not address topics related to peri-implant pathologies but included the truncated term in the abstract; these were later discarded. Secondly, self-citation can be a source of bias in bibliometrics. However, among the 100 articles, the self-citation rate was 2.57%, lower than that for general medical articles (5.97%) (25).

Another limitation was the fact that that some authors have had multiple affiliations during their career. When analysing productivity by country, only the last affiliation was taken into account. China’s ranking is attributable to Niklaus Lang having worked at the University of Hong Kong.

## Conclusions

This is the first analysis of the most-cited articles on peri-implantitis published in journals indexed in Web of Science. The most cited articles on peri-implant pathologies were published in high-impact journals. The most cited articles were reviews and the most-cited authors also published in fields other than dentistry, such as periodontics. Sweden and Switzerland were the top-ranked countries for almost all parameters studied, as also seen in other bibliometric studies on implantology.
